# Supplementary figures and images for: Natural variation in the consequences of gene overexpression and its implications for evolutionary trajectories
Source: eLife. 2021 Aug 2;10:e70564. doi: 10.7554/eLife.70564 (PMC8352584; doi:10.7554/eLife.70564)

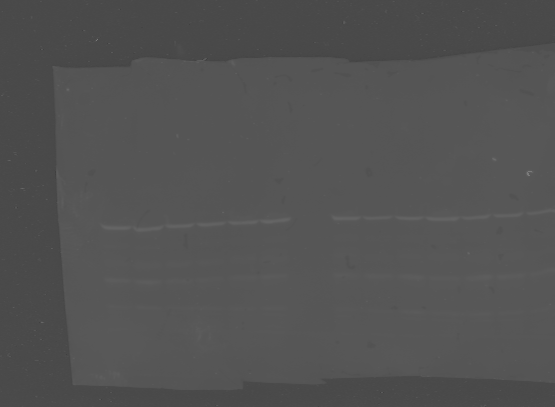

Supplement: Figure 4—figure supplement 1—source data 1. [file elife-70564-fig4-figsupp1-data1.tiff.zip › Figure 4 figure supplement 1 source data 1.tiff]

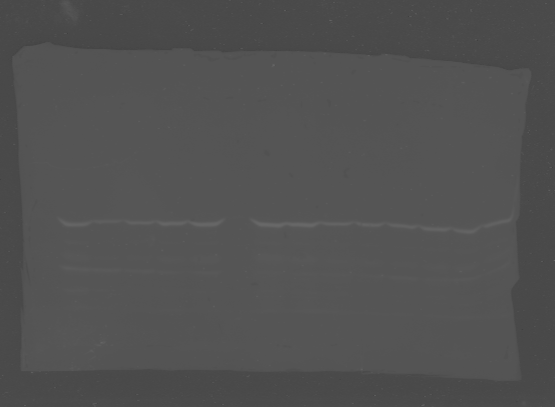

Supplement: Figure 4—figure supplement 1—source data 2. [file elife-70564-fig4-figsupp1-data2.tiff.zip › Figure 4 figure supplement 1 source data 2.tiff]

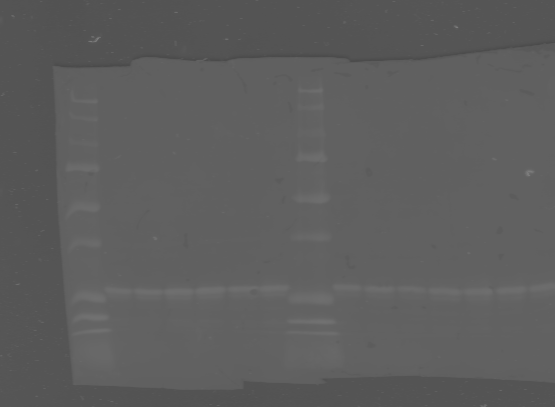

Supplement: Figure 4—figure supplement 1—source data 3. [file elife-70564-fig4-figsupp1-data3.tiff.zip › Figure 4 figure supplement 1 source data 3.tiff]

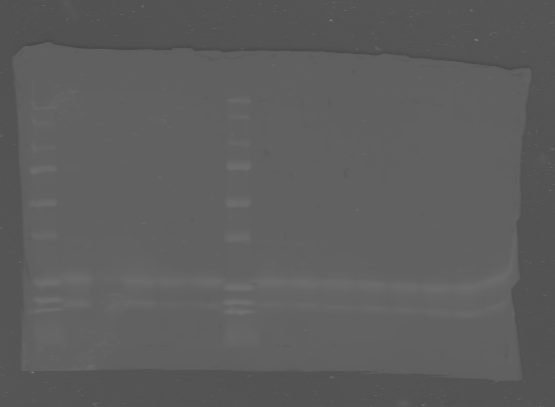

Supplement: Figure 4—figure supplement 1—source data 4. [file elife-70564-fig4-figsupp1-data4.tiff.zip › Figure 4 figure supplement 1 source data 4.tiff]

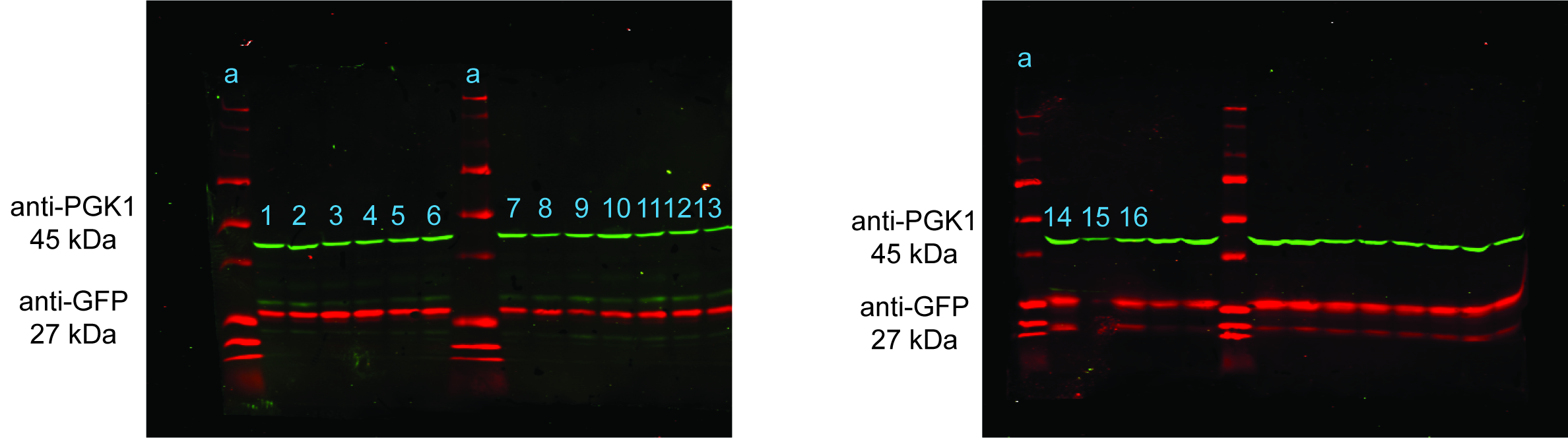

Supplement: Figure 4—figure supplement 1—source data 5. [file elife-70564-fig4-figsupp1-data5.tif.zip › Figure 4 figure supplement 1 source data 5.tif]
